# Comparison of the use of opioids only and pregabalin add-on for the treatment of neuropathic pain in cervical myelopathy patients: a pilot trial

**DOI:** 10.1038/s41598-020-65108-8

**Published:** 2020-05-15

**Authors:** Jong-myung Jung, Chun Kee Chung, Chi Heon Kim, Seung Heon Yang, Yunhee Choi

**Affiliations:** 1 Department of Neurosurgery, Spine Center, Gachon University Gil Medical Center, Incheon, Republic of Korea; 2 Department of Neurosurgery, Seoul National University Hospital, Seoul National University College of Medicine, Seoul, Republic of Korea; 3 Division of Medical Statistics, Medical Research Collaborating Center, Seoul National University Hospital, Seoul National University College of Medicine, Seoul, Republic of Korea

**Keywords:** Neurological disorders, Pain, Clinical trial design

## Abstract

Among patients with cervical myelopathy who were diagnosed with neuropathic pain (NP) by the LANSS test, the study participants were randomly assigned to one of the two study groups. The participants in one study group received opioids only, while those in the other group received opioids and pregabalin. Thirty-nine patients were analyzed in the study (20 patients in the opioid-only group and 19 in the pregabalin add-on group). The LANSS, neck pain, and arm pain scores in the pregabalin add-on group improved significantly compared with those in the opioid-only group after the first 4 weeks (*p* = 0.005, 0.001 and 0.035, respectively), but there was no significant difference between the two groups during the next 4 weeks (*p* = 0.615, 0.377 and 0.716, respectively). There was no significant difference in the neck disability index and EuroQol-5Dimension scores after four weeks and eight weeks of follow-up. Adverse events were reported by four patients (20.0%) in the opioid-only group and five patients (26.3%) in the pregabalin add-on group (*p* = 0.716). However, over time, the occurrence of side effects and dropouts increased in the pregabalin add-on group. This exploratory pilot study suggests that pregabalin add-on treatment is more efficient than the use of opioids alone at the beginning of NP treatment in cervical myelopathy patients. However, prescribing pregabalin add-on treatment for more than four weeks should be done cautiously.

## Introduction

Neuropathic pain (NP) is defined as pain caused by a lesion or dysfunction of the central or peripheral nervous system^1,2^. Although some medications are available for NP, including opioids, tramadol, antidepressants, and different antiepileptic drugs (AEDs), a systematic review suggests that considering the balance between efficacy and tolerability, pregabalin may be recommended as a first-line treatment for NP^3–5^. Pregabalin is an AEDs prescribed for the treatment of various diseases such as epilepsy, spasticity, anxiety, and NP^6^. The use of AEDs in the treatment of NP is based on several similarities in the pathophysiology and biochemical mechanisms of epilepsy and NP. It is frequently used for the treatment of several NP syndromes, and it has been approved by the Food and Drug Administration for the treatment of post-herpetic neuralgia, fibromyalgia, diabetic peripheral neuropathy, and spinal cord injury^7–11^. In post-herpetic neuralgia, significantly more patients in the pregabalin group were responders (> or =50% decrease in mean pain scores from baseline to endpoint) than in the placebo group^11^. Additionally, pregabalin significantly reduced weekly mean sleep interference scores. Health-related quality-of-life measurements using the SF-36 Health Survey have demonstrated improvement in the mental health, bodily pain and vitality domains in pregabalin groups. Pregabalin is safe and effective for decreasing pain associated with diabetic peripheral neuropathy and also improves mood, sleep disturbances, and quality of life^9,10^. In central NP associated with spinal cord injury, the mean endpoint pain score was found to be lower in the pregabalin group than in the placebo group^8^. Pregabalin was associated with improvements in sleep disturbances and anxiety, and more patients reported global improvement at the endpoint in the pregabalin group. The off-label use of pregabalin for various pain syndromes, especially for NP, is widespread. However, there is little literature supporting this practice, and the cost is likely astronomical. On 1 April 2019, pregabalin and gabapentin were reclassified as class C controlled substances in the UK. The reclassification was prompted by a growing number of deaths associated with misuse of the two drugs: the number of deaths linked to pregabalin increased sharply from four in 2012 to 136 in 2017, and those related to gabapentin rose from eight in 2012 to 59 in 2016^12^. In the previous five years, prescribing pregabalin has increased by 350% and gabapentin by 150%^13^.

Spinal cord injury guidelines recommend pregabalin as the first choice of first-line medications for the reduction of NP intensity among people with spinal cord injury^14^. Cervical myelopathy is the most common form of spinal cord injury in adults in North America, with an incidence of 76 per million^15^. The present definition of cervical myelopathy is both the presence of long-tract signs and high signal changes on T2 MRI in the cervical spinal cord^16,17^. There is also little information on the effect of pregabalin in patients with NP in cervical myelopathy under routine clinical practice.

The current clinical trial was developed to provide pilot data regarding the efficacy of pregabalin add-on treatment for NP in cervical myelopathic patients and to provide enough positive data to support the conduct of a subsequent large definitive randomized placebo-controlled trial.

## Materials and Methods

### Study design and patient groups

This study was a randomized, open-label, pilot trial. The study was approved by an institutional review board (H-1609-118-795) and was registered with clinicaltirals.gov (NCT-03618589). Informed consent was obtained from the study participants after explaining the off-label use of pregabalin and its possible adverse effects.

No previous studies have reported the effect of pregabalin on NP in cervical myelopathy patients. Since this was a pilot study, the calculation of sample size was not performed. It was determined by the possible number of patients within the study period. The study participants were selected with strict criteria (Table 1). Patients with current or prior gabapentin or pregabalin use, chronic use of narcotic pain medication and antidepressants, a history of addiction or substance abuse and significant neurological deficits were excluded. The study participants were randomly assigned to one of the two study groups. Randomization was computer-generated. The participants in one study group received opioids only, while those in the other received opioids and pregabalin. Patients in the opioid-only group received only opioids (5 mg of oxycodone three times a day) for eight weeks. Patients in the pregabalin add-on group received opioids (5 mg oxycodone three times a day), 75 mg pregabalin twice a day for the first week (150 mg/day), 150 mg pregabalin twice a day (300 mg/day) for the second week, and 300 mg pregabalin twice a day (600 mg/day) for the subsequent six weeks. The overall duration of the treatment was eight weeks. Most of the recruited patients were postoperative patients. Therefore, we could not discontinue opioid medications until the diagnosis of NP. Hence, we decided to examine the additional value of pregabalin. Additionally, the reason behind how we specified opioid medication as oxycodone was that it was most widely used postoperatively in our hospital. The dosage was also decided as the most commonly prescribed postoperative dosage in our hospital.

**Table 1:** Inclusion criteria and exclusion criteria of the present study.

Inclusion Criteria
1. between the ages of 18 to 80 years
2. Among patients with cervical myelopathy, patients with neuropathic pain (LANSS pain scale ≥ 12)
**Exclusion Criteria**
1. Current or prior gabapentin or pregabalin use
2. Chronic use of narcotic pain medications
3. Chronic depression or the use of antidepressants
4. History of addiction and substance abuse
5. Presence of significant motor deficits, and bowel and bladder dysfunction
6. hypersensitivity, history of angioedema, and congestive heart failure
**Dropout Criteria**
1. Worsening neurological signs and symptoms
2. Unacceptable side effects such as dizziness, somnolence, ataxia, cognitive impairment, edema, and myalgia

Abbreviations: LANSS, Leeds assessment of neuropathic symptoms and signs.

The study participants completed the baseline questionnaires (including sensory testing) before taking the medicine. The effects of treatment were evaluated in three areas: pain, disability, and health-related quality of life. The primary outcome, pain changes, was assessed using the Leeds assessment of neuropathic symptoms and signs scale (LANSS) and visual analogue scale (VAS) for the neck and arm. The LANSS pain scale is used to screen for the presence of pain of neuropathic origin because of its high sensitivity and specificity^18^. The LANSS scale has two parts; a pain questionnaire with five items (testing thermal sensation, autonomic changes, dysesthesia, and paroxysmal and evoked pain) and a sensory component assessing allodynia and altered pin-prick threshold (two items). Skin sensitivity was examined by comparing the painful area with a contralateral or adjacent nonpainful area for the presence of allodynia and an altered pin-prick threshold. Allodynia was assessed by investigating the response to the light stroking of cotton wool across the nonpainful area followed by the painful area. The altered pin-prick threshold was determined by comparing the response to a 23-gauge (blue) needle mounted inside a 2 ml syringe barrel placed gently onto the skin in the nonpainful followed by the painful areas. If the LANSS pain scale score was ≥ 12, then neuropathic mechanisms were likely to contribute to the patient’s pain. In this study, only patients with a LANSS pain scale score of 12 points or more were included. The secondary outcomes of changes in disability level and patient satisfaction with treatment were evaluated by using the neck disability index (NDI) and EuroQol-5Dimension (EQ-5D) 5 L, respectively. The EQ-5D evaluates patient-perceived health^19^. The EQ-5D comprises five dimensions: mobility, self-care, usual activities, pain/discomfort, and anxiety/depression. The scores of the five items can be used to calculate a utility index that ranges from −0.6 to 1.0, where a higher score means better patient health. After completing the 4-week and 8-week medication regimens, the study participants visited the outpatient clinic and completed the questionnaires.

### Statistical analysis

The Mann-Whitney U test was used to compare pain, disability, and health-related quality of life between the two groups. The Wilcoxon signed-rank test was used to analyze changes in pain, disability, and health-related quality of life within each group. The Bonferroni correction was utilized to compare the clinical results, which included scores for the LANSS, VAS-N, VAS-A, ODI, and EQ-5D, between both groups at each time point. A generalized linear mixed-effects model was applied to analyze the clinical outcomes. The fixed effects were group, time, the interaction between group and time, and confounding factors such as age, sex, weight, smoking status, diagnosis, and the baseline measurements corresponding to the outcomes for each linear mixed model. A two-sided *p*-value of <0.05 was considered significant. Data analysis was performed using SPSS for Windows, version 22 (SPSS Inc., New York, NY).

## Results

### Study participants and baseline characteristics

**Figure 1:**
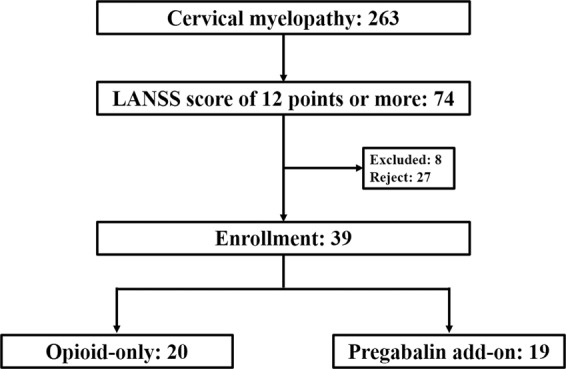
Flowchart of the study participants. LANSS, Leeds assessment of neuropathic symptoms and signs.

We screened 263 cervical myelopathy patients for the study between November 2017 and June 2019 (Fig. 1). Of these, 74 patients had a LANSS score of 12 points or more. Eight patients did not meet the study eligibility criteria and were excluded from the study. The most common reasons for exclusion were current or prior pregabalin/gabapentin use and current use of potent opioids. Twenty-seven patients declined to participate in the study, the majority due to personal preference for avoiding participation in a blinded trial. Finally, 39 patients were analyzed for the study: 20 in the opioid-only group and 19 patients in the pregabalin add-on group.

The baseline characteristics of the study participants are summarized in Table 2. There were no significant differences in age, sex, weight, smoking status, or the cause of cervical myelopathy (diagnosis) between the two groups. Baseline LANSS, VAS for neck pain (VAS-N), VAS for arm pain (VAS-A), NDI, and EQ-5D scores were also not significantly different between the two groups (*p* = 0.525, 0.202, 0.853, 0.733, and 0.446; respectively).

**Table 2:** Demographic and clinical characteristics.

	Opioid-only (n = 20)	Pregabalin add-on (n = 19)	*p*-value
Age	57.5 ± 12.7	52.8 ± 11.4	0.237
Sex (male:female)	11: 9	12: 7	0.748
Weight (kg)	69.2 ± 11.3	65.1 ± 12.2	0.285
Smoking, n (%)	4 (20.0%)	5 (26.3%)	0.716
Diagnosis			0.725
CSM	10 (50.0%)	10 (52.6%)	
OPLL	6 (30.0%)	7 (36.8%)	
Spinal cord tumor	2 (10.0%)	2 (10.5%)	
Spinal cord injury	2 (10.0%)	0 (0.0%)	
Baseline LANSS	15.9 ± 2.0	16.4 ± 3.4	0.525
Baseline VAS-N	7.9 ± 2.2	8.7 ± 1.8	0.202
Baseline VAS-A	8.1 ± 1.9	8.2 ± 1.8	0.853
Baseline NDI	20.9 ± 8.9	21.8 ± 8.1	0.733
Baseline EQ-5D	0.57 ± 0.19	0.62 ± 0.19	0.446
Duration of symptoms (months)	2.9 ± 1.4	2.6 ± 1.1	0.430

Abbreviations: CSM, cervical spondylotic myelopathy; OPLL, Ossification of posterior longitudinal ligament; LANSS, Leeds assessment of neuropathic symptoms and signs; VAS-N, visual analogue scale for neck pain; VAS-A, visual analogue scale for arm pain; NDI, neck disability index; EQ-5D, EuroQol-5Dimension.

The values represent the means ± the standard deviations.

### Treatment responses

The baseline and treatment responses of the opioid-only and pregabalin add-on groups measured in terms of the pain, disability, and satisfaction associated with treatment are presented in Table 3. The primary outcome measure, the LANSS score, was significantly improved in the opioid-only and pregabalin add-on groups after eight weeks (*p* < 0.001). The treatment effect (difference between the opioid-only group and the pregabalin add-on group) was −3.9 ± 1.3 (*p* = 0.005) at the 4-week follow-up and −5.8 ± 1.5 (*p* = 0.003) at the 8-week follow-up. The VAS-N and VAS-A scores improved after 8 weeks in both groups, with significant differences. The treatment effect of the VAS-N was statistically significant at the 4-week and 8-week follow-ups (*p* = 0.001, and 0.001, respectively). The treatment effect of the VAS-A was also statistically significant at the 4-week follow-up (*p* = 0.035), but there was no significant difference at the 8-week follow-up (*p* = 0.125). The NDI and the EQ-5D scores improved in both groups without significant differences at the 8-week follow-up, and the treatment effect was not significant at the 4-week and 8-week follow-ups.

**Table 3:** Clinical outcomes during the study periods.

		baseline	4 weeks	8 weeks	*p*-value^‡^
LANSS	Opioid-only	15.9 ± 2.0	13.1 ± 4.1	10.7 ± 1.4	**<0.001**
	Pregabalin add-on	16.4 ± 3.4	10.2 ± 2.4	6.9 ± 2.4	**<0.001**
	Treatment effect^*^	—	−3.9 ± 1.3	−5.8 ± 1.5	
	(*p*-value^†^)	—	**0.005**	**0.003**	
VAS-N	Opioid-only	7.9 ± 2.2	5.1 ± 1.6	3.3 ± 1.2	**<0.001**
	Pregabalin add-on	8.7 ± 1.8	4.0 ± 1.0	1.9 ± 0.5	**<0.001**
	Treatment effect^*^	—	−2.3 ± 0.6	−2.9 ± 0.8	
	(*p*-value^†^)	—	**0.001**	**0.001**	
VAS-A	Opioid-only	8.1 ± 1.9	5.4 ± 1.5	3.6 ± 1.3	**<0.001**
	Pregabalin add-on	8.2 ± 1.8	4.0 ± 1.3	2.2 ± 1.2	**<0.001**
	Treatment effect^*^	—	−1.2 ± 0.5	−1.5 ± 1.0	
	(*p*-value^†^)	—	**0.035**	0.125	
NDI	Opioid-only	20.9 ± 8.9	16.8 ± 6.8	13.5 ± 3.8	0.107
	Pregabalin add-on	21.8 ± 8.1	16.3 ± 6.7	14.0 ± 5.2	0.112
	Treatment effect^*^	—	0.6 ± 3.3	0.6 ± 1.7	
	(*p*-value^†^)	—	0.847	0.706	
EQ-5D	Opioid-only	0.57 ± 0.19	0.68 ± 0.08	0.76 ± 0.08	0.150
	Pregabalin add-on	0.62 ± 0.19	0.71 ± 0.12	0.77 ± 0.11	0.117
	Treatment effect^*^	—	0.02 ± 0.05	0.03 ± 0.02	
	(*p*-value^†^)	—	0.645	0.138	

Abbreviations: LANSS, Leeds assessment of neuropathic symptoms and signs; VAS-N, visual analogue scale for neck pain; VAS-A, visual analogue scale for arm pain; NDI, neck disability index; EQ-5D, EuroQol-5Dimension.

*linear mixed-effects model; †between-group difference; ‡time difference.

The values represent the means ± the standard deviations. Boldface type indicates statistical significance.

The interval changes of the LANSS, VAS-N, VAS-A, NDI, and EQ-5D scores are summarized in Table 4. Analysis of the changes showed that the LANSS score in the pregabalin add-on group improved more significantly than that in the opioid-only group during the first 4 weeks, but there was no significant difference between the two groups over the next four weeks (*p* = 0.005 and 0.615, respectively). The VAS-N score in the pregabalin add-on group decreased more significantly than that in the opioid-only group during the first 4 weeks, but there was no significant difference between the two groups over the next four weeks (*p* = 0.001 and 0.377, respectively). The VAS-A score in the pregabalin add-on group also decreased more significantly than that in the opioid-only group during the first 4 weeks (*p* = 0.035), but the VAS-A score in the opioid-only group decreased more than that in the pregabalin add-on group over the next four weeks (*p* = 0.716). The NDI score improved during the first four weeks and over the next four weeks in both groups, but there was no significant difference between the two groups (*p* = 0.847, 0.279, respectively). The EQ-5D score also improved in the first four weeks and over the next four weeks in both groups, but there was no significant difference between the two groups.

**Table 4:** Interval changes of the LANSS, VAS-N, VAS-A, NDI, and EQ-5D scores after the first 4 weeks and during the next 4 weeks.

	Opioid-only group	Pregabalin add-on group	*p*-value
**LANSS**			
From baseline to 4 weeks	2.9 ± 3.5	6.2 ± 2.8	**0.005**
From 4 weeks to 8 weeks	2.4 ± 4.0	3.3 ± 2.5	0.615
**VAS-N**			
From baseline to 4 weeks	2.8 ± 1.7	4.7 ± 1.6	**0.001**
From 4 weeks to 8 weeks	1.8 ± 1.3	2.2 ± 1.0	0.377
**VAS-A**			
From baseline to 4 weeks	2.8 ± 1.5	4.1 ± 1.6	**0.035**
From 4 weeks to 8 weeks	1.9 ± 1.9	1.7 ± 1.5	0.716
**NDI**			
From baseline to 4 weeks	4.1 ± 8.7	5.5 ± 6.8	0.847
From 4 weeks to 8 weeks	3.3 ± 6.7	2.3 ± 5.9	0.279
**EQ-5D**			
From baseline to 4 weeks	0.11 ± 0.14	0.08 ± 0.14	0.645
From 4 weeks to 8 weeks	0.09 ± 0.08	0.06 ± 0.12	0.765

Abbreviations: LANSS, Leeds assessment of neuropathic symptoms and signs; VAS-N, visual analogue scale for neck pain; VAS-A, visual analogue scale for arm pain; NDI, neck disability index; EQ-5D, EuroQol-5Dimension.

The values represent the means ± the standard deviations. Boldface type indicates statistical significance.

### Adverse events

**Figure 2:**
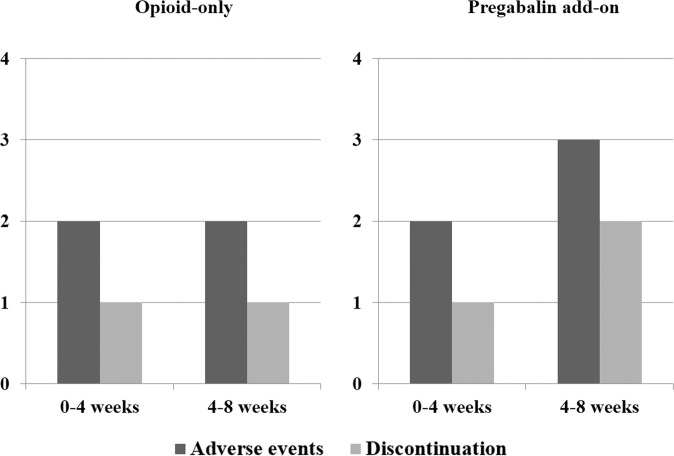
The incidences of treatment‐induced adverse events and discontinuation in the opioid-only and pregabalin add-on groups.

The incidences of adverse events and discontinuation are described in Fig. 2. Adverse events were reported by four patients (20.0%) in the opioid-only group and five patients (26.3%) in the pregabalin add-on group (*p* = 0.716). Two patients with dizziness, one patient with nausea, and one patient with constipation were reported in the opioid-only group. In the pregabalin add-on group, two patients complained of somnolence, 2 of dizziness and 1 of dry mouth. A total of 2 (10.0%) patients in the opioid-only group and 3 (15.8%) in the pregabalin add-on group discontinued treatment due to adverse events (*p* = 0.661).

## Discussion

The currently recommended first-line pharmacological treatments for NP include anticonvulsants (gabapentin and pregabalin), tricyclic antidepressants (amitriptyline and nortriptyline), serotonin-noradrenalin reuptake inhibitors (duloxetine and venlafaxine), and opioids^20–24^. In a systemic review, in only two of seven studies reporting useful efficacy data was amitriptyline significantly better than placebo^25^. For nortriptyline, only one study reported the primary outcome of people with at least a 50% reduction in pain^26^. For venlafaxine, four of the six studies reported some positive benefit^27^. In the largest study by Rowbotham, 56% of participants achieved at least a 50% reduction in pain intensity versus 34% of participants in the placebo group^28^. However, this study was subject to significant selection bias. In a survey of opioids for NP in adults, at least 50% pain intensity reduction was reported in three studies (265 participants, 110 events)^29^. Using a random-effects analysis, 70/132 (53%) had at least 50% pain relief with opioids and 40/133 (30%) with placebo. Opioid drugs, including oxycodone, are commonly used to treat NP, and they are considered effective by some professionals. A systematic review assessed the analgesic efficacy of oxycodone for chronic NP in adults^30^. There was only very low-quality evidence that oxycodone was of value in the treatment of painful diabetic neuropathy or postherpetic neuralgia. There was no evidence for other NP conditions, including cancer-related neuropathy, central NP, type II complex regional pain syndrome, human immunodeficiency virus neuropathy, painful diabetic neuropathy, phantom limb pain, postherpetic neuralgia, postoperative or traumatic NP, spinal cord injury, and trigeminal neuralgia. However, there have been no studies regarding NP in cervical myelopathic patients.

In this study, among patients with cervical myelopathy, the proportion of NP patients with a LANSS pain score of 12 points or more was higher than expected (28.1%). However, a large number of patients (more than 36.5%) refused to participate in the study. Therefore, we could only recruit 39 patients for 20 months. Our study population was composed of a small but relatively homogeneous target group of patients with NP and cervical myelopathy.

In Table 3, pregabalin add-on treatment could be misinterpreted as being more effective than the use of opioids only, but examining the changes over time could lead to different conclusions. Table 4 showed that the pregabalin add-on group had significantly better LANSS scores than the opioid-only group until four weeks after the start of the medication (*p* = 0.005). However, there was no significant difference in the change in LANSS scores between the two groups over the next four weeks (*p* = 0.615). The VAS-N and VAS-A scores also showed similar changes after medication. These results suggest that the use of pregabalin add-on treatment for NP in patients with cervical myelopathy is useful in the early stages (approximately four weeks), but after that, the effect is negligible. The present study also analyzed the satisfaction of patients (EQ-5D) after medication. In this study, the opioid-only group and the pregabalin add-on group did not show significant differences in the changes in EQ-5D scores at the beginning of the treatment (*p* = 0.645) or over the next four weeks (*p* = 0.765); the same results were observed for the NDI. It is noteworthy that pregabalin add-on treatment was effective for reducing pain but did not significantly affect disability or satisfaction. It is thought that the NDI and EQ-5D scores did not improve because cervical myelopathy did not improve. The modified Japanese Orthopaedic Association score has been found to have a moderately strong negative correlation with the NDI^31^. Another study has shown a decrease in EQ-5D scores in patients with cervical myelopathy^32^.

In the opioid-only group, during the first four weeks, two patients complained of side effects, one of whom stopped taking the medicine due to dizziness. During the next four weeks, two patients complained of side effects, one of whom stopped taking the medicine due to nausea. There was no difference in the occurrence of side effects or dropouts between the two periods. In the pregabalin add-on group, during the first four weeks, two patients complained of side effects, one of whom stopped taking the medicine due to somnolence. During the next four weeks, three patients complained of side effects, two of whom stopped taking the medicine due to dizziness and dry mouth. Over time, the occurrence of side effects and dropouts increased. In a nationwide study of opioids, 0.60% of patients experienced severe opioid-related adverse events^33^. In a systematic review of pregabalin for acute and chronic pain, side effects were reported to occur in 3–5% of cases^34^. The duration of therapy is one of the predictive factors for adverse events associated with pregabalin administered for NP^35^. Although it is not possible to give a precise period, long-term administration of pregabalin may cause more side effects. Although some studies have reported that opioids are ineffective for NP, other studies show that NP can be improved by opioids^36–38^. Even though the treatment with pregabalin was of short duration, and the patients were followed for only a brief period, the results of this study are meaningful.

Our study had several limitations, including a small sample size, which resulted in low statistical power. Another limitation was that our study did not evaluate pharmacological effects. Moreover, there was also no systematic assessment of the tolerability of treatment. However, the high percentage of patients who completed treatment and the low frequency of dropouts due to side effects means that opioids could represent a useful treatment option for patients with NP and cervical myelopathy. The third limitation was the open‐label design of the study, introducing possible sources of bias in terms of the patients. A double-blind and double-dummy design could minimize such bias. However, due to ethical issues and practical situations, such models could not be used. The fourth limitation was that cervical myelopathy is a condition of chronic progressive atraumatic spinal cord compression that, over time, may cause a decline in physical function and quality of life. All of the diagnoses included in this study (cervical spondylotic myelopathy, ossification of posterior longitudinal ligament, spinal cord tumor, and spinal cord injury) can progress even after surgical treatment. However, all of these conditions would have had a similar impact on the primary endpoint. The fifth limitation of our study was that repeated use of a questionnaire could impact its validity. Finally, patients in neither of the groups were taking pregabalin alone.

Despite its limitations, this study is important for several reasons. First, we were able to randomly assign patients with NP and cervical myelopathy to receive treatment with opioids only or pregabalin add-on treatment since the study recruited patients before the use of pregabalin and gabapentin had become common. To our knowledge, this study is the first randomized, open-label, pilot trial in which the impact of pregabalin add-on treatment is examined explicitly for NP in cervical myelopathy patients. However, the now increasingly common prescription of these medications by primary care providers may preclude a large-scale study in the future, or a wash-out period may be needed for the discontinuation of pregabalin. Second, the results of this study will lead to a larger, well-controlled, randomized clinical trial regarding the efficacy of pregabalin for the treatment of NP in cervical myelopathy patients before such use becomes permanently engrained among pain physicians.

We demonstrated that pregabalin add-on treatment was effective for the treatment of NP in the short term. There remains a persistent need to evaluate the excessive practice of pregabalin use in a large proportion of patients with NP and cervical myelopathy. Replication with a larger sample size is recommended to confirm the results of this study.
